# The comparison of selenium and lead accumulation between contaminated muddy and sandy sediments from four estuaries along the Persian Gulf: effect of grain size

**DOI:** 10.1007/s10653-018-0078-z

**Published:** 2018-04-18

**Authors:** Mehdi Hosseini, Nooshin Sajjadi

**Affiliations:** 1 Young Researchers and Elite Club, South Tehran Branch, Islamic Azad University, Tehran, Iran; 2 Department of Environmental Science, Faculty of Marine Science and Technology, Islamic Azad University, North Tehran Branch, Tehran, Iran

**Keywords:** Selenium, Lead, Geoaccumulation index, Enrichment factor, Persian Gulf

## Abstract

Persian Gulf is one of the most important water sources in the economically developed south part of Iran, and metal pollution is a major concern for the Gulf. The bioavailability and distribution of selenium (Se) and lead (Pb) between muddy and sandy sediments from four estuaries along the Persian Gulf were analyzed. The geoaccumulation index and enrichment factor for metals and correlation between particles size with metals concentration were studied. The average concentration of metals in sediment was ranged 0.08–1.14 µg/g for Se and 0.32–4.37 µg/g for Pb in all estuaries, with the highest concentrations in Musa estuary. The results showed there was positive correlation between particles size of sediment with metals concentration. The highest of metal concentration was absorbed in silt (< 63 μm) sediment, followed by extremely fine sand (63–125 μm), fine sand (125–250 μm), medium sand (250–500 μm) and coarse sand (500–1000 μm), respectively. The organic matter and carbonate in the muddy sediment are higher than sandy sediment, and they had high specific storage capacity for accumulation of heavy metals. The result of Pearson correlation (*r*) for organic matter and metal was 0.78 for Se and 0.67 for Pb, and for carbonates and metal was 0.54 for Se and 0.61 for Pb. The values of EF in all estuaries show that the enrichment of sediment by heavy metals was by anthropogenic activities such as discharge of petrochemical and oil industrial. Geoaccumulation index indicates that the sediment in the Ahmadi and Ghanam estuaries was unpolluted to moderately polluted, while in the Arvand river is moderately to strongly polluted, and in the Musa estuary is extremely polluted.

## Introduction

Heavy metals are a natural part of the earth’s crust. Heavy metals enter the sea usually through riverine influx (after weathering and erosion of rocks), atmospheric deposition (dust particles, e.g., from volcano’s) and anthropogenic activities (de Mora et al. 2004). Humans add both to the riverine disposition (waste water of factories) and atmospheric deposition (cars and factories). Heavy metals are stable and can not be broken down, which makes it easy for them to accumulate in the environment (Raeisi Sarasiab et al. 2014). The amount of toxic metal near the surface of many of the world’s oceans has tripled as the result of our polluting activities and a new study has found, with potentially damaging implications for marine life as the result of the accumulation of the toxic metal (Hosseini et al. 2014b; Raeisi Sarasiab et al. 2014).

Heavy metals introduced in the marine ecosystem are mostly concentrated in coastal areas, near densely populated and industrialized regions. Heavy metals are usually associated with particles (Chen 2002; Aktaruzzaman et al. 2014). These particles are often very small and can therefore stay in solution for a very long time. Nevertheless, they will end up in the sediments; therefore, concentrations in the sediments are often 10–100 times higher than those in solution. In the sediments, these particles may form an important secondary source of contamination, even after the primary source has disappeared (Bellucci et al. 2003; Asibor et al. 2015). Sediments are important sinks for various pollutants such as heavy metals and also play a useful role in the assessment of metals contamination and pollution in the marine environment (Ikem et al. 2003). In addition, metal-contaminated sediments may release metals into the overlying water column and thus pose a risk to aquatic life and ecosystems (Alagarsamy 2006; McCready et al. 2006: Hosseini et al. 2014b).

Cation exchange capacity is based on the surface area of sediment grain particles available for binding cations, such as hydrogen (H^+^) and free metal ions (e.g., Mn^+2^). Sediments with a high percentage of small grains, such as silt and clay, have high surface-to-volume ratios and can adsorb more heavy metals than sediments composed of large grains, such as sandy (Domagalski 2001; Jakkapan et al. 2015). Total organic carbon is added to sediments primarily through the decomposition of plant and animal matter. Organic carbon can directly adsorb heavy metals from solutions applied to sediments (Vicente-Martorell et al. 2009). However, it can also contain heavy metals accumulated by plants that have been exposed to contaminated sediment during their lifetimes (Raeisi Sarasiab et al. 2014). Nonetheless, high percentages of organic matter and/or small grains in sediment are generally associated with reduced heavy metal bioavailability and toxicity (Hosseini et al. 2015). Therefore, there are high correlation between heavy metal pollution and grain size in sediments, and the pollution level in sediment with small grain (clay) is higher the compare with large grains (sand) (Ramesh et al. 1990; Kishe and Machiwa 2003; Ikem et al. 2003; Vicente-Martorell et al. 2009; Raeisi Sarasiab et al. 2014; Hosseini et al. 2014b; Vaezi et al. 2015).

Lead is accumulating in the surface layers of the seas faster than in the deep ocean, as we pour the element into the atmosphere and seas from a variety of sources, including mines, coal-fired power plants and sewage (Vaezi et al. 2015). Lead is toxic to humans and marine life and accumulates in our bodies over time as we are exposed to sources of it. Lead can be widely dispersed across the globe when it is deposited in water and the air, the authors said, so even parts of the globe remote from industrial sources can quickly suffer elevated levels of the toxic material (Pirrone et al. 2001).

Selenium pollution is a worldwide phenomenon and is associated with a broad spectrum of human activities, ranging from the most basic agricultural practices to the most high-tech industrial processes (Ramesh et al. 1990; Mooraki et al. 2009). Consequently, selenium contamination of aquatic habitats can take place in urban, suburban, and rural settings alike-from mountains to plains, from deserts to rainforests, and from the Arctic to the tropics. Human activities that increase waterborne concentrations of selenium are on the rise, and the threat of widespread impacts to aquatic life is greater than ever before (Domagalski 2001). Important sources of selenium contamination in aquatic habitats are often overlooked by environmental biologists and ecological risk assessors due to preoccupation with other, higher priority pollutants, yet selenium may pose the most serious long-term risk to aquatic habitats and fishery resources (Bellucci et al. 2003). Failure to include selenium in the list of constituents measured in contaminant screening/monitoring programs is a major mistake, both from the hazard assessment aspect and from the pollution control aspect. Once selenium contamination begins, a cascade of bioaccumulation events is set into motion which makes meaningful intervention nearly impossible (Kishe and Machiwa 2003).

The measurement of trace metal concentrations and distribution in the marine environment leads to better understanding of their behavior in the aquatic environment and is important for detecting sources of pollution (Ikem et al. 2003; Pirrone et al. 2001; Vicente-Martorell et al. 2009). Studies on metal concentrations in the surface sediment from Persian Gulf have been carried out, but limited data are available on the distribution of selenium and lead concentration in this area. Therefore, this study, provide new information on the distribution and concentration of selenium and lead in the muddy and sandy sediments collected four estuaries along the Persian Gulf in the south Iran. Also, we studied the correlation between grain size, organic carbon, nitrogen, carbonate, pH and metal concentration in sediment.

## Materials and methods

### Sample collection

**Fig. 1:**
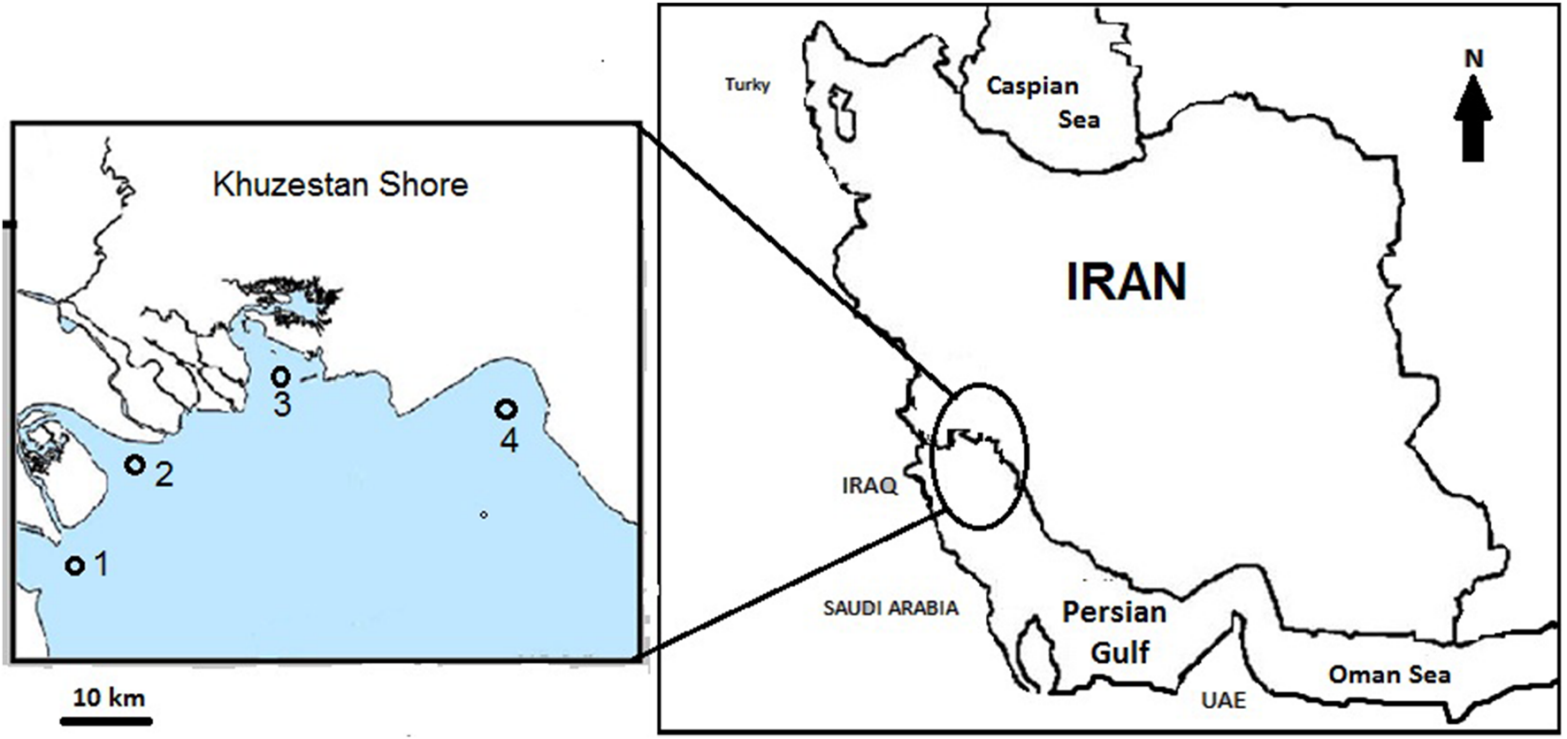
Map of Persian Gulf and study area

Sediment samples obtained from four estuaries including, Arvand river, Ghanam, Musa and Ahmadi estuaries along Khuzestan Shore, north part of the Persian Gulf (Fig. 1). Surface sediments were collected in April of 2014 by a Van Veen Grab. Subsamples were taken from the uppermost layer of the sediment taking care to minimize contamination. Surface sediments (0–5 cm) were sectioned and stored in pre-combusted glass jars in a freezer (− 20 °C) until analysis. Before analysis, sediments were freeze-dried and ground to achieve homogeneity. For determining the relationship between grain size and metal contents, the grain size of surface sediments was measured using a Beckman Coulter laser particle size analyzer (Model LS 13 320). Briefly, 20 mL deionized water was added to 1 g of freeze-dried sediment in a beaker. After soaking for 24 h, the sediment was subjected to vortex mixing for 5 min to disaggregate loosely attached aggregates. Neither organic matter nor carbonate was removed for the laser grain size analysis (Ramesh et al. 1990; Bellucci et al. 2003). The size range of detection for this analyzer is from 0.02 to 1000 μm. Mineral-specific surface area was measured after the sediment was muffled overnight at 350 °C, using the one-point BET method on a Quantachrome Monosorb analyzer. The all samples were divided into two samples such as sandy (> 63 μm) [extremely fine sand (63–125 μm), fine sand (125–250 μm), medium sand (250–500 μm) and coarse sand (500–1000 μm)] and muddy (4–63 μm) sediment.

### Chemical and microbiological analyses

Total organic carbon (TOC) in sediment was measured by a CHN elemental analyzer, after the carbonates were removed by acid fuming in a sealed container with concentrated HCl. The grain size of surface sediments was measured using a Beckman Coulter laser particle size analyzer (Model LS 13 320). Briefly, 20 mL deionized water was added to 1 g of freeze-dried sediment in a beaker. After soaking for 24 h, the sediment was subjected to vortex mixing for 5 min to disaggregate loosely attached aggregates. Neither organic matter nor carbonate was removed for the laser grain size analysis. The size range of detection for this analyzer is from 0.02 to 2000 μm. Mineral-specific surface area was measured after the sediment was muffled overnight at 350 °C, using the one-point BET method on a Quantachrome Monosorb analyzer (Alagarsamy 2006).

The pH of the sediments was measured in distilled water with a 1: 2.5 sediment/solution ratio (Domagalski 2001). The content of organic matter in each sample was determined using loss on ignition. Portions of 5.0 g oven-dried sediment from each site were heated at 450 °C for 20 h. Each sample was re-weighted after being cooled to determine level of organic content by the loss in weight of dry sediment. The environmental parameters of sediments collected from all station are shown in Table 1.

**Table 1:** Organic carbon (OC), carbonate (CaCO_3_), nitrogen (N) and pH in muddy and sandy sediment from four station

Sediment type	Environmental parameters	Station			
		S1	S2	S3	S4
Muddy	Organic carbon (OC)	0.23	0.17	0.09	073
Sandy		0.18	0.11	0.10	0.54
Muddy	Carbonate (CaCo_3_)	0.63	0.32	0.21	0.85
Sandy		0.45	0.28	0.17	0.73
Muddy	Nitrogen (N)	150	260	450	500
Sandy		170	240	470	550
Muddy	PH	8.1	7.8	8	8.2
Sandy	pH	7.9	7.9	7.8	8.1

S1: Arvand river; S2: Ghanam estuary; S3: Ahmadi estuary; S4: Musa estuary

### Heavy metal analyses

For each sample, a known quantity (1 g) of sediment was digested with a solution of concentrated HClO_4_ (2 mL) and HF (10 mL) to near dryness. Subsequently, a second addition of HClO_4_ (1 ml) and HF (10 ml) was made and the mixture was evaporated to near dryness. Finally, HClO_4_ (1 ml) alone was added and the sample was evaporated until white fumes appeared. The residue was dissolved in concentrated HCl and diluted to 25 ml (Abdolahpur Monikh et al. 2012). Recovery varied between 97.8 and 103%. Metal concentrations were determined by a cold vapor atomic absorption spectrometer Leco AMA-254. The accuracy of the analytical procedures was assessed using the certified reference material BCR-1 and yielded results within the reference value range (Ramesh et al. 1990).

### Enrichment factor (EF) analyses

It is well known that metals originating from the same source generally group together, mainly in silt and clay fractions and enrichment, if it occurs, can be observed by using a normalization procedure that offsets the variability in mineralogy and grain size by establishing the enrichment factor (EF) defined as ratio between the following ratios: the element concentration and the conservative element concentration in the sample and; the element concentration and the conservative element concentration in the background reference values, in this case, the surrounding soil of the dam (Upadhyay et al. 2006). The elements of natural origin that are structurally combined with one or more mineral phases are considered conservative. The main assumption for the application of a geochemical normalization for conservative elements is the existence of a linear relationship between the normalizer and other metals and its concentration should not be anthropogenically altered (Guilherme et al. 2011). In this study, EF was used as a normalizer according to Eq. 1:

1 $${\text{EF}}\, = \,{{\left[ {{{C_{n} } \mathord{\left/ {\vphantom {{C_{n} } {{\text{CS}}_{c} }}} \right. \kern-0pt} {{\text{CS}}_{c} }}} \right]\;{\text{sample}}} \mathord{\left/ {\vphantom {{\left[ {{{C_{n} } \mathord{\left/ {\vphantom {{C_{n} } {{\text{CS}}_{c} }}} \right. \kern-0pt} {{\text{CS}}_{c} }}} \right]\;{\text{sample}}} {\left[ {{{C_{n} } \mathord{\left/ {\vphantom {{C_{n} } {{\text{CS}}_{c} }}} \right. \kern-0pt} {{\text{CS}}_{c} }}} \right]}}} \right. \kern-0pt} {\left[ {{{C_{n} } \mathord{\left/ {\vphantom {{C_{n} } {{\text{CS}}_{c} }}} \right. \kern-0pt} {{\text{CS}}_{c} }}} \right]}}\left( {\text{shale}} \right)$$

According to Idris (2008), if 0.5 < EF < 1.5, the elemental concentration is probably entirely due to crustal or natural weathering origin; values above 1.5 indicate anthropogenic contribution. The higher the EF value the more severe is the anthropogenic contribution (Guilherme et al. 2011).

### Geoaccumulation index (Igeo) analyses

The degree of pollution in sediments can be assessed by the determination of indices such as geoaccumulation index (Igeo). Another approach to assess the contamination level of sediments is the Igeo defined by Muller (1979) according to Eq. 2:

2 $${\text{Igeo}}\, = \,\log_{2} \left( {{{C_{n} } \mathord{\left/ {\vphantom {{C_{n} } {1.5_{x} B_{n} }}} \right. \kern-0pt} {1.5_{x} B_{n} }}} \right)$$

where *C*_*n*_ = concentration of the examined element in the sediment sample; *B*_*n*_ = background of a given element in the geochemical reference. The geoaccumulation index distinguishes six classes of quality for sediments: Igeo < 0 unpolluted; 0 < Igeo < 1 unpolluted to moderately polluted; 1 < Igeo < 2 moderately polluted; 2 < Igeo < 3 moderately to strongly polluted; 3 < Igeo < 4 strongly polluted; 4 < Igeo < 5 strongly to extremely polluted; 5 < Igeo extremely polluted. The class 6 is an open class and comprises all values of the geoaccumulation index higher than 5.

### Statistical analyses

All data were tested for normal distribution with Shapiro–wilk normality test. One-way analysis of variance (ANOVA) followed by Duncan post hoc test was used to compare the data by station. The metal concentration of each sample is expressed in micrograms of metal per gram dry of sediment (µg/g), and a probability of *p* = 0.05 was set to indicate statistical significance.

## Results and discussion

Table 2 shows the mean and comparison of the selenium and lead concentration in muddy and sandy sediment from four estuaries along Khuzestan Shore, north part of the Persian Gulf. According to these data, mean concentrations of selenium were 0.65 ± 0.01 for Arvand river, 0.34 ± 0.04 Ghanam estuary, 0.11 ± 0.05 Ahmadi estuary and 1.14 ± 0.02 for Musa estuary in muddy sediment, and 0.38 ± 0.01 for Arvand river, 0.14 ± 0.03 Ghanam estuary, 0.08 ± 0.01 Ahmadi estuary and 0.87 ± 0.02 for Musa estuary in sandy sediment. There was significant difference in selenium concentration between different stations (*α* < 0.05). The highest concentrations of selenium were detected in sediment of Musa estuary, followed by Arvand river, Ghanam and Ahmadi estuary, respectively. Also, there was significant difference in selenium concentration between muddy and sandy sediment (*α* < 0.05). The concentration of selenium in muddy sediments was higher than sandy sediments.

**Table 2:** Selenium and lead concentrations (µg/g) in sediments collected from muddy and sandy sediment

Sediment type	Metal	Station			
		Arvand river	Ghanam estuary	Ahmadi estuary	Musa estuary
Muddy	Se	0.65 ± 0.01^b^	0.34 ± 0.24^Ns^	0.11 ± 0.05^Ns^	1.14 ± 0.02^a^
Sandy		0.38 ± 0.06^b^	0.14 ± 0.13^Ns^	0.08 ± 0.01^Ns^	0.87 ± 0.12^a^
Muddy	Pb	1.82 ± 0.13^a^	0.64 ± 0.03^b^	0.75 ± 0.05^Ns^	4.37 ± 0.53^a^
Sandy		1.26 ± 0.21^a^	0.32 ± 0.16^b^	0.43 ± 0.44^Ns^	2.94 ± 0.31^a^

^a,b^significant difference between sampling sites

^Ns^not significant difference between sampling sites

The mean concentrations of lead were 1.82 ± 0.03 for Arvand river, 0.64 ± 0.03 Ghanam estuary, 0.75 ± 0.05 Ahmadi estuary and 4.37 ± 0.02 for Musa estuary in muddy sediment, and 1.26 ± 0.01 for Arvand river, 0.32 ± 0.06 Ghanam estuary, 0.43 ± 0.04 Ahmadi estuary and 2.94 ± 0.01 for Musa estuary in sandy sediment. There was significant difference in selenium concentration between different stations (*α* < 0.05). The highest concentrations of selenium were detected in sediment of Musa estuary, followed by Arvand river, Ahmadi estuary and Ghanam, respectively. Also, there was significant difference in selenium concentration between muddy and sandy sediments (*p* < 0.05). The concentration of selenium in muddy sediments was higher than sandy sediments.

Metals concentrations in the sediment with different particle size are presented in Table 3. The mean concentration of Se in the sediments with different grain size was 1.14 µg/g in silt, 0.88 µg/g in extremely fine sand, 0.57 µg/g in fine sand, 0.16 µg/g medium sand and 0.08 µg/g coarse sand, respectively. The mean concentration of Pb was 4.37 µg/g in silt, 2.08 µg/g in extremely fine sand, 1.18 µg/g in fine sand, 0.54 µg/g medium sand and 0.32 µg/g coarse sand, respectively. There is significant difference between different size of sediment (*α* < 0.05), and the order of metal in the different size of sediments was as follows: silt (< 63 μm) > extremely fine sand > fine sand > medium sand > coarse sand.

**Table 3:** Metal concentrations (µg/g) in different particle size fractions

Metal	Particle size fraction				
	Silt (< 63 μm)	Extremely fine sand (63–125 μm)	Fine sand (125–250 μm)	Medium sand (250–500 μm)	Coarse sand (500–1000 μm)
Se	1.14 ± 0.02^a^	0.88 ± 0.24^b^	0.57 ± 0.34^b^	0.16 ± 0.41^c^	0.08 ± 0.11^c^
Pb	4.37 ± 0.23^a^	2.08 ± 0.18^b^	1.18 ± 0.16^b^	0.54 ± 0.07^c^	0.32 ± 0.12^c^

^a,b,c^significant difference between sampling sites

The correlation between the heavy metal content and the grain size of particular fractions raises a question whether the content of metals in the fractions of the smallest grain size actually reflects the state of pollution of the natural environment, as it is assumed, e.g., results of our studies have shown that the contribution of the fractions of the smallest grain size in the sediments is high from all stations. In the sandy samples from all station, the concentration of heavy metal was lower than muddy sediment. Therefore, it can be concluded there was high correlation between heavy metal content and the grain size of sediment. The highest of metal concentration was absorbed in silt (< 63 μm) sediment, followed by extremely fine sand, fine sand, medium sand and coarse sand, respectively.

Ikem et al. (2003) investigated on concentration of heavy metal in sediment from Tuskegee Lake, Southeastern USA. They showed there is a significant correlation between metal concentration and particles size of sediment. This means that reducing the size of the sediment particles cause increase metal concentration. McCready et al. (2006) studied the metallic and organic contaminants in sediments of Sydney Harbour, Australia. Their results showed metal concentrations is higher in muddy sediment than sediment with coarse particles. There is a positive correlation between amount of organic carbon and metal concentration. The amount of organic carbon is more in the muddy sediment; consequently, accumulation of metal is more in this type of deposit (Vicente-Martorell et al. 2009; Hosseini et al. 2014b; Vaezi et al. 2015).

It was obvious that heavy metals were not homogeneously distributed among the various particle fractions, suggesting the influence of sediment texture on the partitioning of heavy metals in the suspended sediments. In general, the concentrations of heavy metals in muddy sediment (silt, < 64 μm) were higher than in the sandy sediments (sand, 64–1000 μm). These might be attributed to the great surface area per unit of mass of the fine particles, which increases the adsorption capacity of these fractions (Ikem et al. 2003). Furthermore, finer suspended sediment particles may accumulate higher concentrations of heavy metals due to the high content of clay minerals, oxides and hydroxides, carbonates, and organic matter, because heavy metal availability is influenced by characteristics of the sediment system such as pH, organic carbon, carbonate and nitrogen content and acid reduction (Domagalski 2001; Kishe and Machiwa 2003).

The best Pearson correlation between chemical characteristics of sediment with metal concentration is showed in Table 4. Organic carbon has a high specific storage capacity for heavy metals (Chen 2002), and Pearson correlation (*r*) was 0.78 (*p* < 0.001) between organic matter and Se and 0.67 (*p* < 0.001) between organic matter and Pb. Results showed that the concentration of metal increased with increasing organic matter present in the sediments. Also, Pearson correlation (*r*) were 0.83 (*p* < 0.001) for organic matter with muddy sediment and 0.55 (*p* < 0.001) for organic matter with sandy sediment; therefore, the organic carbon level in the sediment with small grain was significantly higher than sandy sediments. Ikem et al. 2003 showed there is positive correlation between organic carbon and heavy metal accumulation in the sediments from Southeastern USA. The high correlation between accumulation of heavy metals and organic carbon levels in the sediment from Persian Gulf was reported by some researcher (Karbassi et al. 2008; Abdolahpur Monikh et al. 2012; Hosseini et al. 2014b; Raeisi Sarasiab et al. 2014).

**Table 4:** Correlation matrix of muddy, sandy, organic carbon, carbonate, nitrogen, pH and metal concentrations in sediment samples

	Se	Pb	Muddy	Sandy	OC	CaCo_3_	N	pH
Se	1							
Pb	0.08	1						
Muddy	0.89	0.83	1					
Sandy	0.45	0.52	0.45	1				
OC	0.78	0.67	0.83	0.55	1			
CaCo_3_	0.54	0.61	0.71	0.44	− 0.34	1		
N	0.23	0.43	− 0.25	0.66	− 0.27	− 0.23	1	
pH	0.55	0.69		− 0.72	− 0.14	0.14	0.12	1

*OC* organic Carbon, *CaCo*_*3*_ carbonate, *N* nitrogen

There was high correlation between carbonate and metal concentration in the sediment samples, and Pearson correlation (*r*) was 0.54 for Se and 0.61 for Pb. Also, the correlation was 0.74 between carbonate and muddy sediment and 0.38 between carbonate and sandy sediment. Therefore, the carbonate level in muddy sediment was higher than sandy sediment; this means that increases in the carbonate level cause will be increases in concentration of heavy metals in the sediments. There was positive correlation between pH and accumulation of heavy metal in sediment, (*r* = 0.55 for Se and 0.69 for Pb with pH); therefore, the pH has important role for accumulation of heavy metal in sediment.

Acidic pH condition is known to influence the sorption of metal by organic matter fraction in sediments (Alagarsamy 2006; Ikem et al. 2003). Analysis of pH and percentage of organic carbon present in sediments demonstrate that there were strong correlations between each of these factors and the concentrations of metal. The organic carbon has high ability for sorption of metal in the sediment in condition with high pH (pH > 8) (Ramesh et al. 1990; Pirrone et al. 2001; Qingzhen et al. 2015). Therefore, results showed that positive correlation was carried out between organic carbon percentages, carbonates content and pH with concentration of heavy metal in the sediments, because they have high capacity for absorb of heavy metals from sediment contaminated.

McCready et al. (2006) showed the heavy metals pollution is significantly higher in the muddy sediment, because the organic carbon had high capacity for absorb of heavy metals from sediment, and its levels is high in muddy sediment. Hosseini et al. 2014b reported that organic carbon and carbonate are the important factors for absorption of heavy metal in the sediment. They showed its level in the sediment with small grain (muddy) are higher the compare than larger grain (sandy). Raeisi Sarasiab et al. (2014) studied on heavy metal distribution in contaminated surface sediments from four estuaries, north part of Persian Gulf. They showed there is a significant correlation between organic carbon and carbonate levels with metal concentration. Also, Karbassi and Amirnezhad (2004), Mooraki et al. (2009), Hosseini et al. (2014a, b), Vaezi et al. (2015) and ZareZadeh et al. (2017) have similar results with present study in the sediment contaminated from Persian Gulf, south Iran.

The oxygen level and water flow are other reasons for changes of heavy metal concentration in sediments (Rocha et al. 2011; Tao et al. 2015). Therefore, there are correlation between content heavy metal and level of oxygen and water flow on sediments. A reduction in the amount of oxygen and water flow causes the increase in accumulation of heavy metal, because the flow of water causes to be leaching and carry off the contaminants (Rocha et al. 2011). Also, decrease in the amount of oxygen in sediment causes the increase in the organic matter. Therefore, the level of oxygen and water flow is lower in muddy sediments; consequently, the level of heavy metal is higher than sandy sediments. Bellucci et al. (2003) reported that there is correlation between oxygen level and water flow in sediment with heavy metal accumulation and concentration. Also, Abdolahpur Monikh et al. (2012) and Raeisi Sarasiab et al. (2014) suggested with results of this study.

### Enrichment factor (EF)

The enrichment factor (EF) is a convenient measure of geochemical trends and is used for making comparisons between areas (Guilherme et al. 2011). A value of unity denotes neither enrichment nor depletion relative to the earth’s crust. The anthropogenic impact could be quantified by calculating the enrichment factor. Table 5 shows the enrichment factor for Se and Pb in muddy and sandy sediment from four estuaries. Enrichment factor was detected for Se, a value of 0.45 < EF < 2.13 and for Pb value of 0.55 < EF < 3.21 in muddy sediment.

**Table 5:** Enrichment factor (EF) for Se and Pb in muddy and sandy sediments from different stations

Sediment type	Metal	Station			
		Arvand river	Ghanam estuary	Ahmadi estuary	Musa estuary
Muddy	Se	1.07	0.87	0.45	2.13
	Pb	1.95	0.76	0.55	3.21
Sandy	Se	0.86	0.62	0.21	1.26
	Pb	1.02	0.37	0.24	1.58

Also, enrichment factor was detected for Se 0.21 < EF < 1.26 and for Pb 0.24 < EF < 1.58. This result suggests that traces of Se may be due to crustal materials or natural weathering processes in muddy and sandy sediment for all estuaries expect, for muddy sediment in Musa estuary. In the Musa estuary, enrichment factor was concluded 2.13 in muddy sediment and suggests that indicate anthropogenic contribution. This means that anthropogenic activities can lead to increase in Se concentration in the muddy with sediments.

**Fig. 2:**
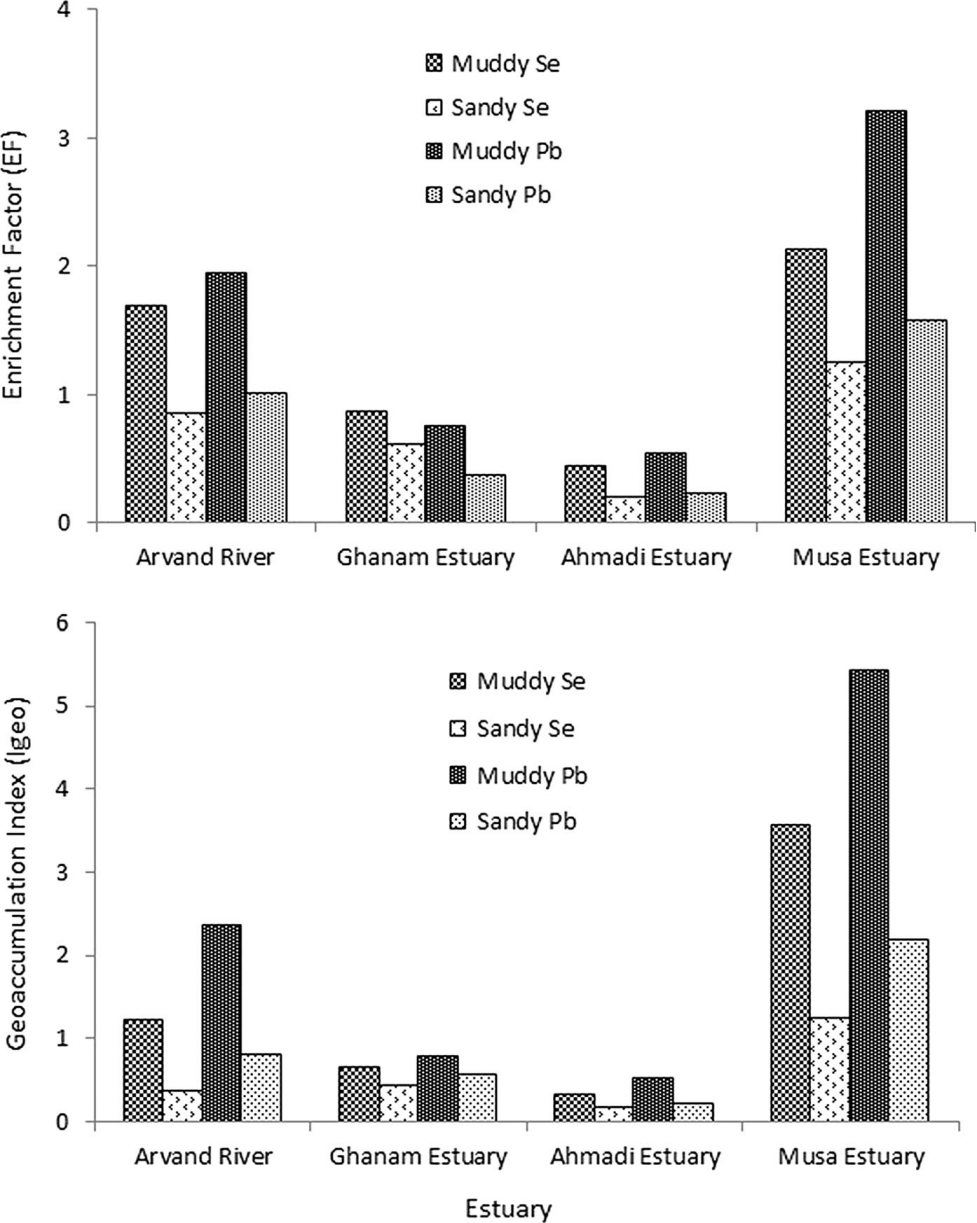
The comparison of geoaccumulation index and enrichment factor between all estuaries

The comparison of enrichment factor between all estuaries is showed in Fig. 2. For Pb, enrichment factor was detected 1.58 in sandy sediment from Musa estuary and in other stations were low than 1.5 (1.5 > EF). This result showed that anthropogenic contribution in this station was higher than other station and high concentration of Pb-related anthropogenic activities. But in muddy with sediment, enrichment factor was detected 1.95 and 3.21 in order for Arvand river and Musa estuary. This means that anthropogenic activities can lead to increase in Pb concentration especially Musa estuary that have highest Pb concentration. In estuarine stations Musa estuary and Arvand river with muddy sediment, which presented EF > 1.5, there is evidence that an important proportion of traces metal is delivered from others sources, suggesting environmental contamination by metal and corresponding to the most contaminated region of estuarine system (Fig. 2).

Nowrouzi and Pourkhabbaz (2014), and Vaezi et al. (2015) showed that enrichment factor for some heavy metal (Pb, Hg, Si and Ni) in sediment from Persian Gulf was higher than 1.5 and the metals sources related to anthropogenic activities such as petrochemical and oil industries. Upadhyay et al. (2006) showed that enrichment factor was between 1.34 < EF < 5.4 for Pb and 1.65 < EF < 6.4 for Se in river Subernarekha, India. Huerta-Diaz et al. (2008) showed that enrichment factor was 1.3 for Pb and 2.5 for Se in coasts of the California. Also, other studies showed that enrichment factor level for Pb and Se in sediment was higher than 1.5 and anthropogenic impact is the main reason for metal pollution (Aktaruzzaman et al. 2014; Asibor et al. 2015; Jakkapan et al. 2015).

### Geoaccumulation index (Igeo)

Concentrations of geochemical background are multiplied each time by the constant 1.5 in order to allow content fluctuations of a given substance in the environment as well as very small anthropogenic influences. Tables 6 shows the Igeo level foe Se and Pb in all estuaries. Igeo concluded in sandy sediments for Se and Pb were unpolluted to moderately polluted in all stations expect for Musa estuary. This station is moderately polluted by Se and moderately to strongly polluted by Pb. In muddy sediment, Igeo in Ghanam and Ahmadi estuaries were unpolluted to moderately polluted by both metal. Igeo in Arvand river was moderately polluted (1.21) for Se and (2.36) moderately to strongly polluted. Musa estuary was detected strongly polluted (3.57) for Se and extremely polluted (5.42) for Pb. Therefore, result shows that Musa estuary with muddy sediment is extremely polluted. Arvand river station with muddy sediments has high concentration of both metal, but metal concentration is low in other estuaries (Fig. 2).

There are different studies that showed geoaccumulation index values for Pb and Se was higher than 5 (strongly polluted) in the sediment from Persian Gulf (Karbassi et al. 2008; Saeedi and Karbassi 2006; Hosseini et al. 2014b; Raeisi Sarasiab et al. 2014; Nowrouzi and Pourkhabbaz 2014;Vaezi et al. 2015; ZareZadeh et al. 2017). Moore et al. (2009) reported that that geoaccumulation index values were 4.5 for Pb in and 2.8 for Se in the Maharlu Aaline Lake, Iran. Geoaccumulation index values calculated 8.68 in East, 0.88 in central and 1.36 in West of Qaroun Lake sediments, Egypt, by El-Sayeda et al. (2015). Also, the geoaccumulation index values for Pb were 2.02 in the southwest of Nigeria (Asibor et al. 2015), and 3.42 for in the Gulf of Thailand (Jakkapan et al. 2015). Finally, the results of all studies in the Persian Gulf showed that sediments were strongly polluted with toxic metal such as Se and Pb.

**Table 6:** The geoaccumulation indices (Igeo) for Se and Pb in muddy and sandy sediment from different stations

sediment type	Metal	Station			
		Arvand river	Ghanam estuary	Ahmadi estuary	Musa estuary
Muddy	Se	1.21	0.65	0.32	3.57
	Pb	2.36	0.79	0.51	5.42
Sandy	Se	0.63	0.42	0.17	1.23
	Pb	0.81	0.57	0.21	2.18

### The comparison between estuaries

The comparison of Se and Pb concentration in all estuaries in present study showed that Musa estuary has accumulated high concentration than the other estuaries. Also, the enrichment factor and geoaccumulation index for Se and Pb in Musa estuary were higher than the other estuaries; therefore, this estuary is extremely polluted by Se and Pb. The Musa estuary is near port of Imam Khomeini that located in the northwest of the Persian Gulf where ships and vessels traffic and the existence of several industries especially petrochemical industries caused the influx of various organic and non-organic contaminants such as heavy metals into the ecosystem (Hosseini et al. 2014b). Musa estuary is surrounded by more than 19 petrochemical units such as chlor-alkali plant and superphosphate plant. The Musa estuary is the nearest creek to Mahshahr City, petrochemical units, and constructions of PETZONE (Mooraki et al. 2009). Therefore, Musa estuary receives pollutants from the different types surrounding areas and heavy metal concentration in this station is higher than other stations. Finally, heavy metal in this estuary was sourced from petrochemical activities and not from background geological sources.

## Conclusions

In present study, accumulation and distribution of Se and Pb in the muddy and sandy contaminated sediments from four estuaries along the Persian Gulf were studied. The enrichment factor and geoaccumulation index for metals and also the relationship between grain size, chemical characteristics and metal pollution level in the sediment were investigated. The results showed the mean concentration of metals in surface sediment was ranged 0.08–1.14 µg/g for Se and 0.32–4.37 µg/g for Pb in the all estuaries along the Persian Gulf. The result of Pearson correlation (*r*) showed there are high positive correlation between heavy metal pollution and grain size in sediments, and the pollution level in sediment with small grain (silt and clay) is higher the compare with large grains (sandy). Also, the results showed there was high correlation between organic matter and carbonates in the sediment with heavy metals pollution and they had high specific storage capacity for accumulation of heavy metals. The calculated EF for Se and Pb showed that sources of metals are anthropogenic activities in all estuaries, and not from background geological sources. The calculated Igeo for metals showed that estuaries are polluted with Se and Pb, but Musa estuary is extremely polluted and the important pollution sources are related to petrochemical units and oil industries around the Persian Gulf.
